# F174. OBESITY AND BRAIN INTEGRITY IN SCHIZOPHRENIA AND BIPOLAR DISORDER: DIVERGENT PATTERNS OF WHITE MATTER MICROSTRUCTURE DAMAGE IN A TRANSDIAGNOSTIC APPROACH

**DOI:** 10.1093/schbul/sby017.705

**Published:** 2018-04-01

**Authors:** Ramiro Reckziegel, Raffael Massuda, Leticia Czepielewski, Monise Costanzi, Rodrigo Spinz, Isadora Remus, Clarissa Gama

**Affiliations:** 1 Hospital de Clínicas de Porto Alegre - HCPA; 2 Universidade Federal do Parana - Brazil; 3 Federal University of Rio Grande do Sul; 4 Hospital Presidente Vargas - Brazil

## Abstract

**Background:**

Obesity is associated with both structural and functional changes of the central nervous system, and is frequent in psychiatry settings. The increased prevalence of obesity in schizophrenia (SCZ) and bipolar disorder (BD) is associated with illness severity, functioning impairment and cognitive deficits. It cannot be attributed to biases inherent in treatment-seeking samples, given that this association is detectable even in drug-naïve patients. Diffusion tensor imaging (DTI) analyses of major brain fibers in both disorders show shared abnormalities of white matter. DTI has been employed as a highly sensitive tool to investigate microstructural changes in white matter structure. While gray matter alterations in obesity point to a consistent reduction with increasing body mass index (BMI), volumetric changes in white matter are more complex and less conclusive. Fractional anisotropy (FA) is the most commonly used parameter as it is the best estimate of fiber integrity as well as axonal and myelin degeneration, and has been reported an association with BMI in depressed BD patients, but not explored in SCZ nor in comparison with a control group (CTR). The aim of this study was to analyze the relationship between obesity and brain alterations assessed by DTI in SCZ, BD and CTR.

**Methods:**

In one-hundred fifty (N=150) individuals (SCZ:49; BD:35; CTR:66) were administered clinical rating scales, collected sociodemographic data and submitted to magnetic resonance imaging (MRI) acquisition in a 1.5 T machine. Linear regression models were performed independently for each group in order to test the relationship of BMI on each brain fiber FA, using gender, age and years of disease for the patients as covariates.

**Results:**

The mean BMI was different among groups (F(143)6.533; P= .002), higher in BD group (BD 29.69 ± 6.55; CTR: 25.54 ± 4.25; SCZ: 26.42 ± 6.02). In BD, the model that predicted FA in the left cingulate gyrus endings was significant controlling for covariates (F(4,21)= 3.273; p = .031; Adj. R^2^= .384), with a main effect of BMI (t=-2.870; p= .009; β=- .531). For SCZ and CTR groups, we did not find significant models to predict brain fiber FAs from BMI controlling for covariates.

**Discussion:**

BMI was associated with reduced FA in cingulate gyrus in BD, implying that obesity may play a role in microstructure damage in the limbic system. These findings are in consonance with the literature and may be related with processing of emotional and cognitive responses disrupted in BD. Conversely, it did not predicted FA in SCZ or CTR connection bundles, possibly because of the lower BMI levels in these groups. Also, we were not able to control for treatment adherence, a variable correlated with both white matter integrity and weight gain. At last, obesity appears to be correlated with white matter microstructure in a heterogeneous and disease specific course depending on the underlying psychopathology, showing association with impairment in BD but not SCZ and CTR. Further studies are needed to explore the role of treatment in the interpretation of these findings.

